# Genetic Diversity and Conservation of the Prespa Trout in the Balkans

**DOI:** 10.3390/ijms141223454

**Published:** 2013-11-28

**Authors:** Patrick Berrebi, Christelle Tougard, Sophie Dubois, Zhaojun Shao, Irene Koutseri, Svetozar Petkovski, Alain J. Crivelli

**Affiliations:** 1 Institut des Sciences de l’Evolution, UMR 5554 CNRS/UM2/IRD, Université Montpellier 2, cc065, Place Eugène Bataillon, Montpellier cedex 05 34095, France; E-Mails: christelle.tougard@univ-montp2.fr (C.T.); sophie.dubois@gmail.com (S.D.); zhjshao@gmail.com (Z.S.); 2 Society for the Protection of Prespa, Agios Germanos, Prespa 530 77, Greece; E-Mail: i.koutseri@spp.gr; 3 Society for the Investigation and Conservation of Biodiversity and the Sustainable Development of Natural Ecosystems-BIOECO, Briselska 12, Skopje 1000, FYR of Macedonia; E-Mail: svetozar@unet.com.mk; 4 Station Biologique de la Tour du Valat, Le Sambuc, Arles 13200, France; E-Mail: a.crivelli@tourduvalat.org

**Keywords:** *Salmo peristericus*, microsatellites, sequencing, Prespa Lake, *Salmo trutta* complex

## Abstract

The Balkans are known to have a high level of biodiversity and endemism. No less than 15 taxa have been recorded in salmonids of the *Salmo* genus. Among them, the Prespa trout is found in only four river systems flowing into Lake Macro Prespa, three in the Former Yugoslav Republic of Macedonia and one in Greece. This is the first comprehensive survey of all streams located within the Macro Prespa Basin, encompassing the whole taxon range. A large genetic sample of 536 Prespa trout was collected mainly between 2005 and 2007. The sampling included 59 individuals from the Golema river system, 93 from the Kranska, 260 from the Brajcinska, 119 from the Agios Germanos, and five individuals from the lake itself. These specimens were analyzed with six microsatellite markers and by sequencing the mitochondrial control region. Nuclear data were examined through multidimensional analysis and assignment tests. Five clusters were detected by assignment: Golema, Kranska, Brajcinska upstream, Rzanska Brajcinska tributary and Brajcinska downstream. Most of these river systems thus hosted differentiated Prespa trout populations (with past gene flows likely dating before the construction of dams), except Agios Germanos, which was found to be composed of 5% to 32% of each cluster. Among the five trout individuals from the lake, four originated from Kranska River and one was admixed. Supported parsimonious hypotheses are proposed to explain these specificities. Conservation of this endemic taxon should take these results into account. No translocation should be performed between different tributaries of the lake and preservation of the Brajcinska populations should address the upstream-downstream differentiation described.

## 1. Introduction

The Balkans have a high level of species richness and endemism, and have thus been classified as a European biodiversity hotspot [1,2]. The Balkans were considered to be a refuge area during the Pleistocene glaciations, acting as crossroads on account of their geographic position and latitude [3,4].

As an example of this high level of endemism in salmonids, particularly in the *Salmo* genus, no less than 15 taxa have been recorded and considered as valid species by Kottelat and Freyhof [5]. Among them, the Lake Macro Prespa trout (*Salmo peristericus*, [6]) is considered to be an endemic taxon. Its taxonomic status has been largely discussed since it was identified and described in the Brajcinska river system in the Former Yugoslav Republic (FYR) of Macedonia based on its ornamental spots, body depth, gill raker number and anal fin position [5,6]. Its inclusion in the *S. trutta* complex is still controversial. The present consensus taxonomy of the *Salmo* genus considers the AD (Adriatic origin), DA (Danubian), MA (marble trout), ME (Mediterranean) and AT (Atlantic) lineages as geographic subgroups within *S. trutta.* Like most Balkan trout taxa [7], *S. peristericus* and *S. letnica*, sampled for this study, belong to the AD lineage [8,9]. Only *S. ohridanus* is considered to be a distinct species [10]. For the first time, Snoj *et al*. [8] analyzed Prespa trout populations in the Brajcinska, Kranska and Golema river systems in the FYR of Macedonia and in the Agios Germanos system in Greece, together with several Albanian *S. trutta* populations. Only one haplotype of mitochondrial DNA (mtDNA) control region (CR) was detected in the Prespa tributaries, recognized as ADcs1. This haplotype is included in the *S. trutta* complex and belongs to the Adriatic geographic subgroup (AD; [11,12]) in fact widespread in the northern Mediterranean region [12]. According to Cortey *et al*. [13], ADcs1 is an ancestral haplotype and likely the basis of the overall AD subgroup diversity.

A recent morphological analysis of Delling [14] gave *S. peristericus* a species status, mainly distinguishable by its low gill raker number and slender body as compared to *S. trutta*.

The Prespa trout taxon lives in only four river systems flowing into Lake Macro Prespa from the north and east: three in the FYR of Macedonia (Golema, Kranska and Brajcinska river systems) and one in Greece (Agios Germanos river system). The Agios Germanos river system naturally flowed into Lake Micro Prespa, a southern smaller water body, but was diverted to Lake Macro Prespa in 1936 [15]. This species is seldom observed in Lake Macro Prespa (during this study only 5 individuals were captured by professional fishermen between 2006 and 2011).

The Prespa trout was first reported to be endangered due to habitat destruction, illegal fishing, water abstraction and competition with the introduced rainbow trout and other salmonids [16]. More recently, the Prespa trout was also classified as endangered by the IUCN [17]. The status of Prespa trout should be settled because taxonomic issues are relatively important in conservation. The different funding providers or organizations that set rules and laws have restrictions, e.g., the European Community only considers endangered species, not subspecies or geographic forms. The exact taxonomic status can therefore be a key factor for conservation initiatives.

This is the first comprehensive survey of all streams located within the Macro Prespa basin, encompassing the whole taxon range and using both nuclear and mitochondrial markers. A large sample of 536 Prespa trout was collected between 2005 and 2007 (and 2011 in the lake) in all biotopes hosting this taxon. Samples of seven other taxa were also analyzed using the same methods to compare the genetic makeup of the Prespa trout with that of neighboring and distant taxa.

This study describes the Prespa trout structure based on samples of all populations of this low polymorphic taxon, living within this very limited range. Its distribution, together with anthropic disturbances and climate change, explain and confirm its “endangered” status which now requires special care.

## 2. Results

### 2.1. Distinction of the Prespa Trout Taxon

The first part of this microsatellite analysis was aimed at taxonomically recognizing the samples from the Lake Macro Prespa basin. The Prespa trout samples (536 fishes, Table 1) were compared to local and European trout taxa (153 fishes, see Table S1 in Supplementary Information) in order to avoid any misidentification of potentially introduced foreign taxa.

**Figure 1.**
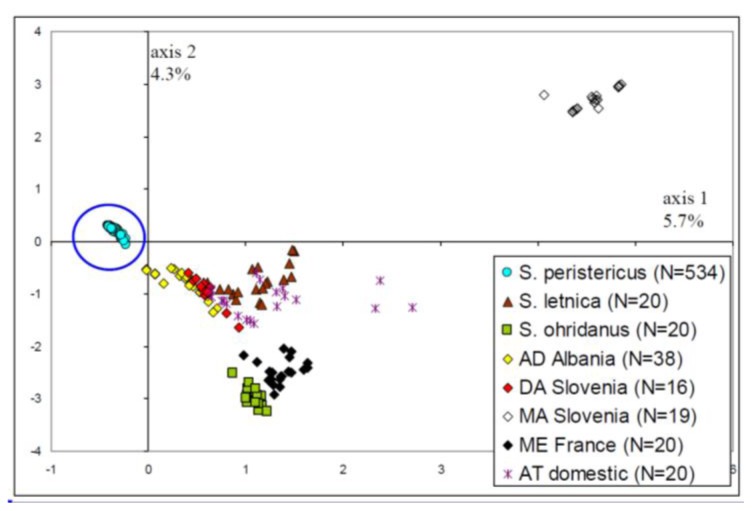
In this multi-taxa global analysis (FCA), all 534 Lake Prespa basin individuals are encompassed in the independent blue ellipse on the left-hand side of the diagram. This taxon is at the top of a triangle with the marble trout (MA) and *S. ohridanus*. Most of the *S. trutta* taxa are inside the triangle. According to the present taxonomy, the AD (Adriatic origin), DA (Danubian), MA (marble trout), ME (Mediterranean) and AT (Atlantic) lineages belong to *S. trutta*; while *S. peristericus* and *S. letnica* belong to the AD lineage. Only *S. ohridanus* is considered as a distinct species.

In the FCA (Figure 1), which involved a multi-taxa global comparison based on microsatellite differentiation, all 536 Lake Prespa basin individuals are included in the independent ellipse on the left-hand side of the diagram. Using microsatellites, Prespa trout can be easily distinguished from the *S. trutta* complex and other close trout taxa.

**Figure 2.**
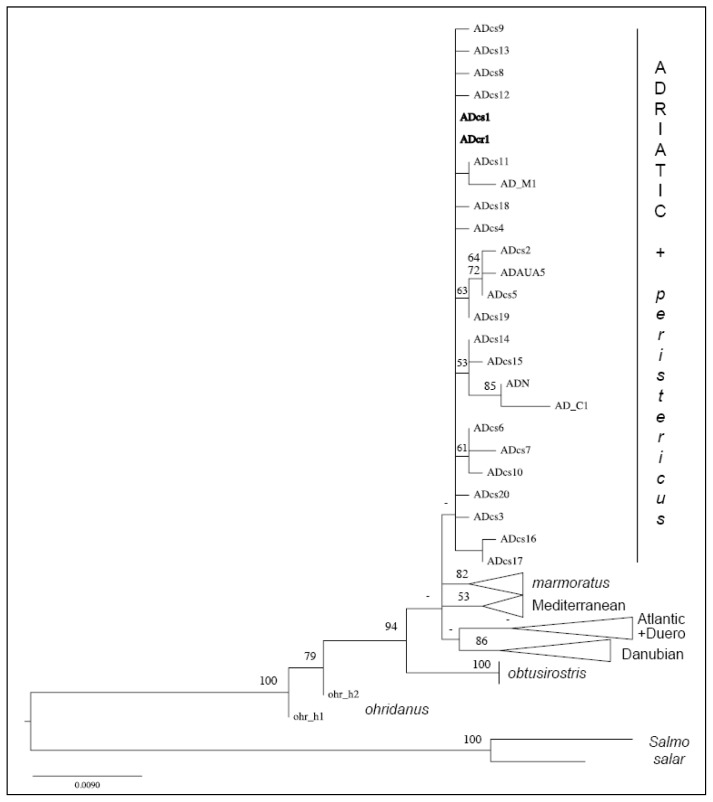
Phylogenetic tree reconstructed using a maximum-likelihood method. The Prespa trout haplotypes are shown in bold.

**Figure 3.**
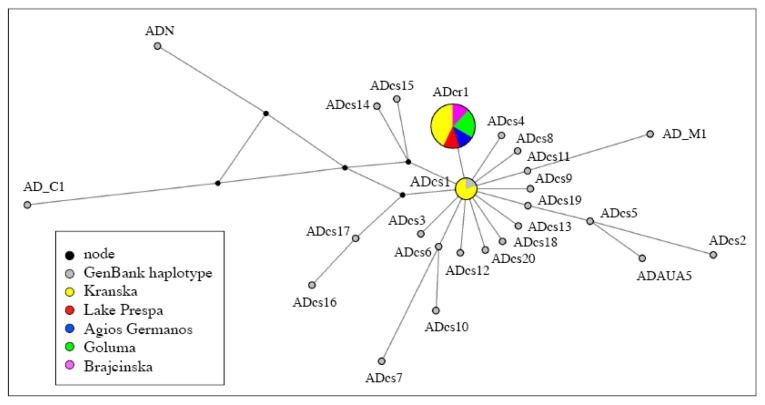
Median-joining network of Prespa trout haplotypes and those of the closest brown trout taxa.

The phylogenetic inference based on the alignment of 989 nucleotides led to the tree topology shown in Figure 2. In this tree, Prespa trout haplotypes (in bold) are clustered with *S. trutta* individuals belonging to the Adriatic subgroup (AD). The Prespa trout sequences only yielded two haplotypes. Eight sequences were identical to haplotype ADcs1 [13]. The 42 remaining sequences differed from ADcs1 by only one substitution, *i.e.*, a T inserted in position 542 of our alignment. This new haplotype was ADcr1, and its original sequence was deposited in the EMBL database under accession number HE863710. In the network including CR haplotypes of the Prespa trout and of AD *S. trutta* (Figure 3), these two haplotypes were closely related (one mutational step) and the central position of the ADcs1 haplotype confirmed its ancestral origin [13].

### 2.2. Biology of the Populations

Panmixia disequilibria are generally observed when migrations occur or when a given sample is composed of several differentiated subgroups (Wahlund effect). Several other causes have been recorded due to selection and adaptation, sometimes linked to the lifecycle. Most of findings of the present tests were insignificant (except Brajcinska River, see Fis Table 2), and most samples were considered to be in Hardy-Weinberg equilibrium.

Hypotheses of errors due to stuttering, of long allele dropout and of null allele were generally rejected by Micro-Checker, except one case (null alleles suspected in sample G4 for locus Ssa197) which was considered as negligible.

Significant linkage disequilibria (after sequential Bonferroni correction) were observed in the Sfo1-Mst85, Oneμ9-Ssa197 and Oneμ9-Mst85 locus pairs, but these were within the lake sample. No linkage disequilibrium was observed in the rivers, which we interpreted as being a lake particularity (e.g., kin relationships) rather than as a marker characteristic.

The Kranska river system seemed to be the most diversified (Hnb = 0.37), followed by Agios Germanos and Brajcinska (0.32 to 0.34); whereas the Leva Reka River (Golema river system) population seemed to be much less polymorphic (0.16). Other parameters (P and A, see Table 2) showed the same trends.

Samples of 10 individuals and more were run for bottleneck detection, so only 26 samples were checked. In the 26 tests, 7 revealed a possible recent bottleneck (*p* < 0.05) in two rivers: (i) in the Kranska river system, in the upstream K2 and K3 samples from 2007 and in the downstream K5 samples for both 2006 and 2007; (ii) in the Agios Germanos river system, only the A2 population showed significant excess heterozygosity in both 2006 and 2007. In the Brajcinska river system, only one case of excess heterozygosity was significant at station B2 (upstream) in 2007, so this finding was considered doubtful.

### 2.3. Population Structure

The Fst data were computed between samples from the same station collected two consecutive summers. This double sampling happened 10 times: at G1, G2 and G3 stations in the Golema river system; K3, K4 and K5 in the Kranska river system; B1, B4, B5 and B7 in the Brajcinska river system and A2 in the Agios Germanos river system. The Fst values obtained were significantly different from zero four times, but never after Bonferroni sequential correction (data not shown). The bi-annual samples from the same station were then pooled for the next analyses, assuming sedentary behavior.

**Figure 4.**
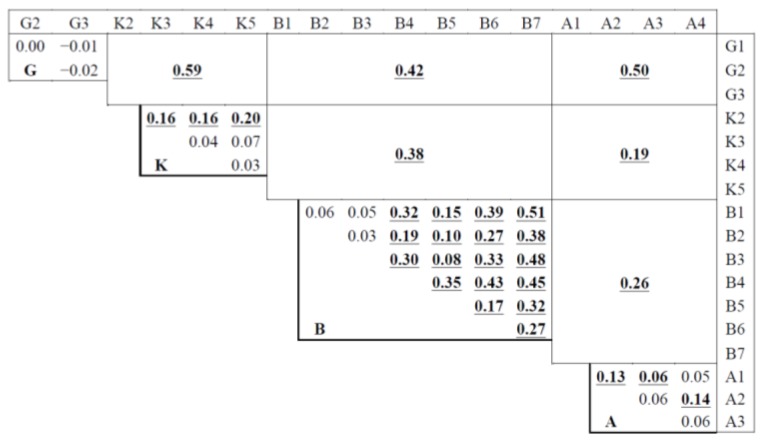
Fst estimated between river systems (large cells) and between stations of the same river system (small cells). The K1 and L samples, limited to five individuals each, were not considered. The significant Fst values after Bonferroni correction are shown in bold and underlined (G = Golema; K = Kranska; B = Brajcinska; A = Agios Germanos).

The different stations within a given river system were compared using Fst (Figure 4). No differentiation was observed among the Golema stations. In the Kranska river system, only the K2 station was differentiated but K3, K4 and K5 stations were genetically similar. K2 is located in one of the upstream tributaries and was probably isolated from the main river. The Brajcinska river system is structured. Most Fst findings were significantly different from zero, except between B1, B2 and B3 stations, which were the most remote from the lake. Finally, the structure within the Agios Germanos river system was unclear: the A1 station seemed to be the most differentiated and the most isolated (located in a tributary).

**Figure 5.**
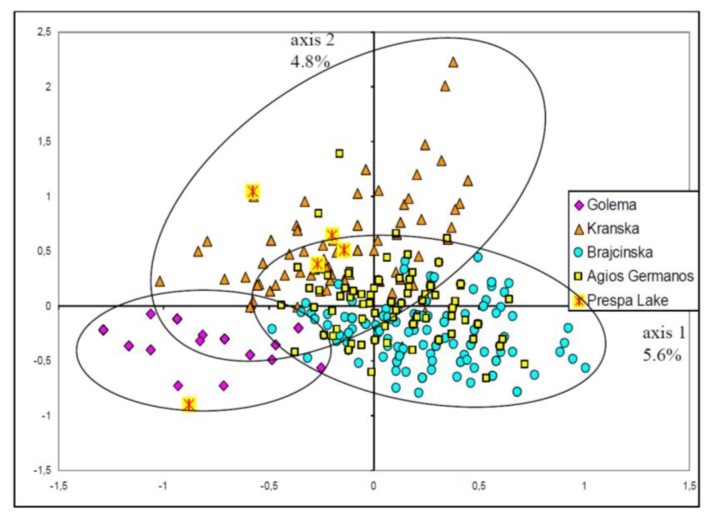
The FCA presented here just concerns the Prespa trout. Weak but clear differentiation was detected between most of the river system samples. However, the genetic diversity was only partially described since no other axes were represented.

The Fst estimations between river systems were all significant (Figure 4). Moreover, the FCA finding, shown in Figure 5, indicated that the sampling was heterogeneous. The envelopes pooling samples from each river system, while partly overlapping, tended to diverge, with the apparent exception of the Agios Germanos samples positioned at the origin of the axes. Most of the five individuals captured in the lake resembled the Kranska type, with one resembling the Golema type. The multidimensional analyses were only indicative and assignment tests would be necessary to confirm the observations.

The whole Prespa trout sample was tentatively divided into 2 to 10 subgroups in the assignment tests. Using the method of Evanno *et al*. [18], *k* is set at 5. Moreover, these assignments made sense up to *k* = 5. For *k* values tested from 6 to 10, the program produced subdivisions within and not between individuals. Table 3 and Figure S1 (Supplementary Information) gives the mean assignment of each sample (or groups of samples) to each cluster.

It was found that the 5 clusters were from Golema (G), Kranska (K), Brajcinska upstream (BU composed of B1, B2 and B3 samples), Brajcinska Rzanska tributary (BR, with only the B4 sample) and Brajcinska downstream (BD composed of B5, B6 and B7 samples). Each river system hosted differentiated Prespa trout populations, except Agios Germanos, which was mainly composed of 72% of the Brajcinska types (B) and 23% of the Kranska type (K). This finding is in agreement with the FCA position of the Agios Germanos trout in the hyperspace (Figure 5).

The five trout individuals from Lake Macro Prespa were highly assigned to the Kranska type (K) except the 2009 individual, which was admixed with the Golema type dominance (Table 3). These results were in close agreement with the FCA findings (Figure 5).

## 3. Discussion

As expected [19], microsatellite crosspriming allowed a large choice of efficient markers. The chosen loci were prepared for several species: *Salvelinus fontinalis* [20], *Salmo salar* [21–23], *S. trutta* [19] and even *Oncorhynchus mykiss* [24].

### 3.1. A Structured Endemic Taxon

The Prespa trout is one of the most endemic trout taxa in the Balkans, currently limited to four tributaries of Lake Prespa covering a distance of about 50 km from north to south. Despite its very limited range, this form is not homogeneous. The Fst calculations revealed clear and strong differentiation between all tributaries (0.38 < *Fst* < 0.59). The assignment tests demonstrated that the Prespa trout could be divided into three well-documented genetic types: Golema (G), Kranska (K) and Brajcinska, composed of three subgroups (BU, BR and BD). Very similar results were obtained with the Bayesian software BAPS. As observed by Bohling *et al*. [25], Structure seems better than BAPS to describe an admixed individual, but it cannot assure that an individual is of pure lineage.

None of the river systems hosts a pure genetic lineage (*sensu* Structure), which could be interpreted as being due to current or recent (before the dam constructions) gene flow between rivers. This gene flow, however, could not lead to the homogenization of the whole taxon.

The particular genetic structure of the Agios Germanos population was surprising. This tributary seemed to include a mixture of 72% of the Brajcinska types (32% of BU, 22% of BR and 18% of BD), 23% of the Kranska type but only 5% of the Golema type. This river, which flowed into another lake (the trout-free Lake Micro Prespa) before 1936, was also probably trout-free until it was artificially connected to Lake Macro Prespa. This connection led to colonization of this new tributary by a mixture of trout, previously inhabiting the lake for feeding purposes, and which were originally derived from several healthy river populations. As a confirmation of the expected scenario, the closer the river mouth is from the Agios Germanos mouth, the higher the participation of its population to the Agios Germanos genetic composition.

### 3.2. A Threatened Species

As the Prespa trout was found to be limited to four river systems flowing into Lake Macro Prespa, this taxon is considered to be vulnerable. The sampled populations were isolated from each other, as suggested by the high Fst estimations (Figure 4). For a given river system, exchanges between the upstream and downstream parts or tributary populations were limited, especially in the Brajcinska river system where a Fst of 0.5 was commonly found. This metapopulation structure could be conducive to local extinction and the high number of stations where no trout were captured (not shown) indicates that natural repopulation would not be easy, due to dam construction and the increase in dry periods. It is likely that the observed gene flow between river systems is a relic of ancient migrations.

Observed and estimated heterozygosities were very low as already observed with allozymes [26] and microsatellites [27]: *i.e.*, between 0.16 and 0.37 in the watersheds considered in this study (Table 2). *S. trutta* populations generally have microsatellite heterozygosity values between 0.6 and 0.8 [28]. The bottleneck investigation generated some information about the recent population size reductions, mainly in the Kranska and Agios Germanos river systems, which could be due to strong spring floods that occur when the snow melts.

The endangered populations concerned the Golema (because of the low polymorphism) and Kranska (because of the low polymorphism due to a past bottleneck) river systems. The Brajcinska and Agios Germanos river systems seemed to have a better capacity to overcome the problem of water reductions and increased human activities.

### 3.3. Management Considerations

The microsatellite data highlighted the fact that Prespa trout is an evolutionary significant unit (ESU) (sensu Waples [29,30]), in agreement with morphology [14], even though its species status has not been confirmed by mtDNA data. Within the Prespa trout populations, we found 5 management units (MUs): Golema (G), Kranska (K), Brajcinska upstream (BU), Brajcinska Rzanska (BR) and Brajcinska downstream (BD). Conservation and management initiatives will have to be considered separately for each of the five MUs. No inter-river system translocations should be carried out to avoid upsetting this structure.

Habitat destruction, frequent poaching and water extraction currently impact all Prespa trout populations. Several dams have been built to divert water for crop irrigation in the summer (June to September). Owing to these dams, the trout cannot reach the lake during the summer and cannot migrate upstream in the autumn during the spawning period, thus putting an abrupt stop to the natural gene flow which occurred in the past. According to tagging experiments [31], the Prespa trout is very sedentary in the rivers, *i.e.*, these fish do not travel more than 100–200 m. When the snow melts in May, however, many 0+ are washed downstream where hundreds of them can be found in the dam lakes and very few can probably go downstream [31]. In the past (before WWII), these 0+ all migrated to the lake where they could grow until reaching sexual maturity before returning to their stream to spawn. The Kranska trout found in the lake could be the result of young fishes that have managed to cross the dams. Young fishes a few centimeters long could cross the dams during overflows or when a flood occurs before the beginning of the irrigation season.

Conservation of multiple and genetically distinct populations is necessary to ensure long-term species survival and ecosystem functioning, but this will require international cooperation.

## 4. Materials and Methods

### 4.1. Sampling Strategy

**Figure 6.**
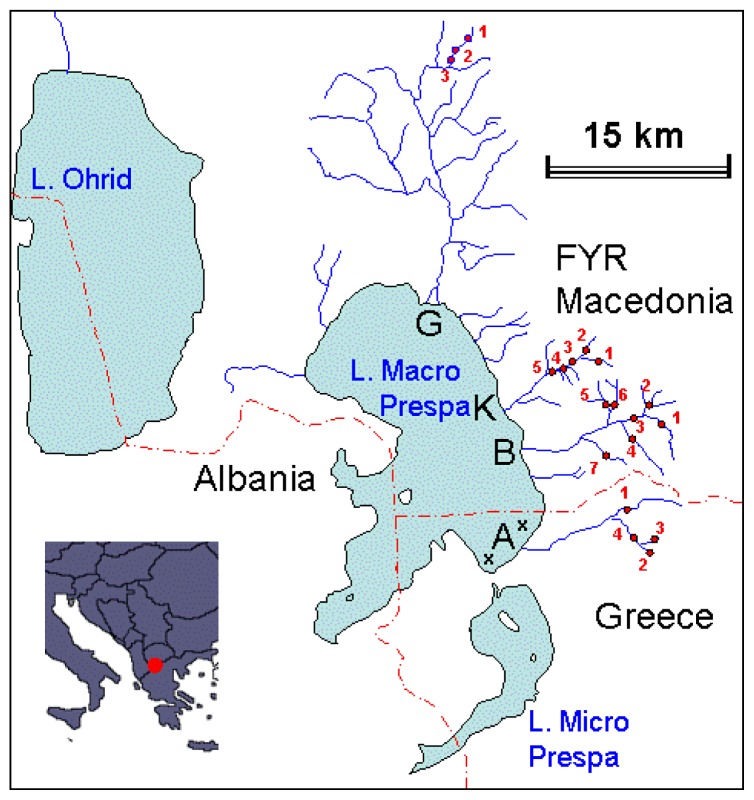
Situation of the 19 sampling localities in the rivers (G = Golema, K = Kranska, B = Brajcinska, A = Agios Germanos) and of the two sampling localities in the lake (x). Trout electrofishing was performed along the entire river system indicated in this figure; the trout lived only at the sampling sites.

Electrofishing was carried out in all tributaries of Lake Macro Prespa mainly during the 2005 to 2007 summer seasons (July and August). A few trout were captured in the lake by professional fishermen using bay nets between 2006 and 2011. The sampling resulted in 5 individuals from the lake itself, 59 from the Golema river system (Leva Reka River), 93 from the Kranska, 260 from the Brajcinska, and 119 from the Agios Germanos (Table 1). Samples were taken from several different sites and at various different dates for each river system. This information is presented in Table 1 and Figure 6.

Most of these fishes were anaesthetized with phenoxyethanol. They were photographed and then a small fin clipping was taken (preserved in 96° ethanol) before they were returned to their river.

For the taxonomic distinction of Prespa trout within the diverse range of trout species found in the Balkans, samples belonging to the Montpellier laboratory tissue collection were analyzed with the same markers. The list of these 153 comparative trout specimens is given in the supplementary document Table S1. Among these individuals, the Albanian trout was previously analyzed by Snoj *et al.* [8] using CR sequences.

### 4.2. DNA Extraction and Genotyping

DNA was extracted from fin tissue samples using the Chelex/proteinase K protocol described by Walsh, Metzger and Higushi [32] and Estoup *et al*. [33]. The six microsatellite markers used in this study, composed of three tetra- and three di-nucleotide loci, were obtained from various sources. Details of each locus are given in Table 4. For each marker, one of the 5′ ends of the two primers was covalently linked to fluorescein, Cy3 or Cy5 labels. Polymerase chain reactions (PCR) were performed in an Eppendorf Mastercycler programmable thermocycler with a 10 μL reaction volume containing 0.2 U of *Taq* polymerase (Sigma-Aldrich, Saint-Quentin Fallavier, France), 1.5 to 2 mM MgCl_2_ (see Table 4), 0.4 mM of each dNTP (Invitrogen, St Aubin, France), 1 μL 10× reaction buffer and 3.75 μM of each primer (Eurofins MWG Operon, Ebersberg, Germany). The thermal cycling conditions were set as follows: initial denaturation (94 °C, 3 min); 30 denaturation (94 °C, 30 s), annealing (15 s at the temperatures given in Table 2 for each locus) and extension (72 °C, 15 s) cycles; and then a final extension (72 °C, 3 min). The PCR products were electrophoresed in 6% denaturing polyacrylamide gels (Bio-Rad, Marnes-le-Coquette, France) and visualized with a FMBIO-II fluorescent imaging system (Hitachi, Yokohama, Japan). Allele sizes were based on a fluorescently labeled ladder 100–600 bp (Promega, Madison, WI, USA), using the FMBIO Analysis 8.0 image analyzer program (Hitachi, Yokohama, Japan). The genotype matrix was then constructed and used as a basis for all of the following statistical analyses.

### 4.3. Mitochondrial DNA Amplification, Sequencing and Data Analysis

CR mtDNA was amplified by PCR using the PST and FST primers [34]. Each 50 μL reaction included 0.4 μM of each primer (Eurofins MWG Operon, Ebersberg, Germany), 0.2 mM of dNTP (2 mM each), 2 mM of MgCl_2_ (25 mM), 10 μL of 5× PCR buffer, 1 U of *Taq* polymerase (GoTaq^®^ Promega, Madison, WI, USA) and 50 ng of genomic DNA. The PCR conditions included initial denaturation (95 °C, 5 min) followed by 30 strand denaturation (94 °C, 1 min), primer annealing (52 °C, 1 min) and DNA extension (72 °C, 1 min) cycles, followed by a final extension (72 °C, 5 min). All PCR amplifications were performed in Eppendorf Mastercycler thermocyclers (Eppendorf, Le Pecq, France). The amplified DNA fragments were run on a 0.8% agarose gel to verify the amplification efficiency. The amplification products were purified and sequenced in both directions to confirm the polymorphic sites by the Macrogen Company (Seoul, Korea).

The CR mtDNA sequences were aligned using the MEGA 5.05 computer program [35]. In order to assign a phylogenetic position to Prespa trout, a phylogenetic analysis was performed, including 50 original CR sequences of Prespa trout from Lake Macro Prespa and all of its tributaries, as well as 151 *S. trutta* (Atlantic, AT; Duero, DU; Mediterranean, ME; Marbled, MA; Danubian, DA; Adriatic, AD), 2 *S. obtusirostris* and 2 *S. ohridanus* CR sequences from GenBank (see Table S1 for sample details). *Salmo salar* was used as the outgroup. The phylogenetic tree was reconstructed using a maximum-likelihood method (ML) with PhyML v2.4.4 [36] under the HKY model [37] with a proportion of invariable sites and a gamma distribution. The nodal robustness was estimated by ML bootstrap percentages after 1000 pseudo-replicates.

The haplotype description of the Prespa trout CR sequences was obtained using DnaSP v5.10.01b [38], while the haplotype relationships and distribution among populations were evaluated with a median-joining network of Prespa trout haplotypes and those of the closest brown trout lineage, as recommended by Mardulyn [39]. This network was constructed with Network v4.5.1.6 (http://www.fluxus-engineering.com/sharenet.htm, [40]).

### 4.4. Microsatellite Descriptive Statistics

The genotype matrix was analyzed using Genetix 4.05 [41]. The following basic analyses were carried out:

A general picture of the trout diversity was obtained through multidimensional analyses (here Factorial Correspondences Analysis [FCA]). This method was based on the microsatellite data only, without any origin information. It was used to confirm that all samples belonged to the Prespa trout taxon, before analyzing intra-taxon genetic differentiation among rivers.
A genetic polymorphism estimation (unbiased estimate of average heterozygosity Hnb [42]) was obtained for each sample and for each river or river system.
The inter-sample or inter-river system differentiations (Fst) and the intra-river panmixia (Fis) were estimated [43]. The significance of the Fis and Fst values was obtained by random permutation procedures (5000 allele permutations within samples for Fis and 5000 individual permutations between samples for Fst).
Linkage disequilibrium screening was performed using the Black and Krafsur method [44] and the significance was determined by permutation tests (5000 permutations).
The sequential Bonferroni correction was applied for multiple tests [45].
Since the same site may have been sampled during the summer of 2005, 2006 and 2007, samples from the same locality collected two years in a row were compared using Fst statistics. The two annual samples were only mixed when the inter-sample Fst could be considered as statistically null.
Finally, a research of errors due to stuttering, long allele dropout and null allele was performed using Micro-Checker[46].

### 4.5. Bottleneck Detection

Since the investigated populations were isolated in small rivers subject to ancient or recent dry out and recent pollution, tests for recent bottlenecks were run using Bottleneck software [47,48] based on the principle that the number of alleles decreases faster than the heterozygosity after a population size reduction. We used the two-phased of mutation model (TPM) including 10% of the stepwise mutation model (SMM), as recommended for microsatellites [49]. The observed and expected heterozygosities were compared using a Wilcoxon sign-rank test, which is relatively powerful and can be used with as few as four polymorphic loci and any number of individuals [48]. The tests were performed sample by sample, but no concatenated samples were considered.

### 4.6. Microsatellite-Based Assignment Tests

Assignment tests were carried out in order to detect differentiated subgroups. This method, using the Bayesian Structure 2.1 program [50] in our case, subdivided the whole sample into *k* = 2 to 10 subgroups characterized by the best genetic equilibrium in terms of panmixia and lower linkage. The admixture ancestry model and correlated allele frequencies were used. A burn-in of 10^5^ iterations followed by 2 × 10^5^ additional Markov Chain Monte Carlo iterations was performed, *i.e.*, slightly over the number recommended by Pritchard *et al.* [50], in order to stabilize assignments among runs. The estimations of the true *k* value (number of biological subgroups in the entire sample—using the Evanno *et al*. [18] method through Structure Harvester[51]—were applied on 20 runs for each *k* value. This automatic estimation was supplemented by the “higher *k* which makes sense” method. By analyzing clustering of the assignment from *k* to *k* + 1 values, the best k is detected when *k* + 1 adds nothing to the comprehension. Generally, *k* + 1 is rejected when adding a new cluster does not highlight a new sample or subsample but adds complexity to several already acceptable clusters. This precaution was taken because, as explained by Pritchard *et al*. [52] and recently by Gilbert *et al*. [53], “selecting the optimal *k* can be quite a subjective procedure and is best inferred when the biology and history of the organism are taken into account”. This analysis was doubled using another Bayesian approach (BAPS) [54] (not shown).

## 5. Conclusions

This case study is characteristic of Balkans endemism. The Prespa trout only inhabits the Lake Macro Prespa tributaries that we sampled. That means that we have here a representation of a whole taxon: the rivers where it is absent have also been unsuccessfully investigated so that the sampling is the distribution of the entire species. Such a taxon is directly threatened by any local human modification and deserves a specific conservation procedure.

Despite its very limited distribution, a clear structure has been evidenced. This basic knowledge is necessary for any future conservation programme.

## Supplementary Information



## Figures and Tables

**Table 1.** Details of samples all around the lake from 2005 to 2011. The numbers of sequenced individuals in parentheses in the last column are from Snoj *et al*. [8] data. In the station abbreviations, a = 2005, b = 2006 and c = 2007.

Station (map)	River system	Tributary	Date of capture	N nDNA	N mtDNA
G1b	Golema	Leva Reka	21 August 2006	10	-
G1c	Golema	Leva Reka	26 July 2007	15	5
G2b	Golema	Leva Reka	21 August 2006	9	(1)
G2c	Golema	Leva Reka	26 July 2007	4	-
G3b	Golema	Leva Reka	21 August 2006	11	(4)
G3c	Golema	Leva Reka	26 July 2007	10	-

K1c	Kranska	*main*	25 July 2007	5	-
K2c	Kranska	Srbino	25 July 2007	16	-
K3b	Kranska	*main*	21 August 2006	10	(1)
K3c	Kranska	*main*	25 July 2007	16	4
K4b	Kranska	*main*	21 August 2006	10	-
K4c	Kranska	*main*	25 July 2007	21	-
K5b	Kranska	*main*	21 August 2006	10	(1)
K5c	Kranska	*main*	25 July 2007	15	-

B1b	Brajcinska	*main*	19 August 2006	10	(1)
B1c	Brajcinska	*main*	23 July 2007	25	-
B2c	Brajcinska	*main*	27 July 2007	31	5
B3c	Brajcinska	Kriva Kobila	27 July 2007	37	-
B4b	Brajcinska	Rzanska	19 August 2006	10	(1)
B4c	Brajcinska	Rzanska	25 July 2007	51	-
B5b	Brajcinska	Drmisar	19 August 2006	10	(1)
B5c	Brajcinska	Drmisar	30 July 2007	28	-
B6c	Brajcinska	Marusica	31 July 2007	16	-
B7b	Brajcinska	Baltanska	12 August 2006	3	(2)
B7c	Brajcinska	Baltanska	30 July 2007	29	-

A1a	Agios Germanos	Siroka	17 August 2005	39	(5)
A2a	Agios Germanos	Gaidouritsa	19 August 2005	10	(3)
A2c	Agios Germanos	Gaidouritsa	3 August 2007	20	5
A3c	Agios Germanos	Gaidouritsa	3 August 2007	28	-
A4c	Agios Germanos	Gaidouritsa	8 August 2007	22	-

L2006	Lake Macro Prespa	*lake*	30 October 2006	2	2
L2009	Lake Macro Prespa	*lake*	21 November 2009	1	1
L2011	Lake Macro Prespa	*lake*	1 August 2011	2	2

**Table 2.** The population genetic polymorphism.

River systems	*N*	*Hn.b.*	*Hobs.*	*P* (0.99)	*Â*	*Fis*
Golema	59	0.16	0.16	0.50	2.00	0.05 NS
SD		0.21	0.21			
Kranska	103	0.37	0.35	0.83	3.17	0.06 NS
SD		0.23	0.22			
Brajcinska	250	0.32	0.23	0.83	3.17	0.29 ***
SD		0.25	0.18			
Agios Germanos	119	0.34	0.32	0.67	2.50	0.01 NS
SD		0.27	0.25			
Lake trout	5	0.64	0.70	1.00	4.00	−0.11 NS
SD		0.26	0.30			

G = Golema; K = Kranska; B = Brajcinska; A = Agios Germanos; L = Lake trout; N = sample sizes; SD = standard deviation; Hn.b. = non biased expected heterozygosity; Hobs. = observed heterozygosity; P (0.99) = polymorphic loci proportion with a maximum allele frequency of 0.99; Â = mean number of alleles by locus; NS = non-significant;

*** = highly significant (*p* < 0.0002).

**Table 3.** When dividing the whole sample into 5 subgroups (lines 1 to 5 of the table), the assignment method produced a comprehensive structure showing a clear link between rivers and partitions. Lines 6 and 7 give the composition of two water bodies and lines 8 to 12 the composition of the 5 trout captured in the lake. For better readability, assignments under 0.05 are not shown and those above 0.50 are in bold.

Sample names	G	K	BU	BR	BD
Golema (G)	**0.92**	-	-	-	-
Kranska (K)	0.05	**0.78**	0.13	-	0.10
Brajcinska upstream (BU)	-	0.07	**0.57**	0.19	0.14
Brajcinska Rzanska (BR)	-	-	0.09	**0.79**	0.06
Brajcinska downstream (BD)	-	-	0.17	0.10	**0.67**

Agios Germanos R.	0.05	0.23	0.32	0.22	0.18
Lake Prespa	0.11	**0.75**	-	0.08	-

Prespa 2006 trout	-	**0.91**	-	-	-
Prespa 2006 trout	-	**0.89**	0.05	-	-
Prespa 2009 trout	0.48	0.12	0.06	0.29	0.06
Prespa 2011 trout	-	**0.90**	-	0.06	-
Prespa 2011 trout	-	**0.95**	-	-	-

**Table 4.** Details of the microsatellite loci used in this study.

Locus	MgCl_2_ (mM)	Annealing temprature (°C)	Allele size (bp) in Prespa trout	Allele size (bp) in *S. trutta*	Reference
Sfo1	2	59	124–138	110–170	[20]
SsoSL311	2	57	122–144	110–196	[21]
Omm1105	2	53	114–218	106–310	[24]
Oneμ9	1.5	60	195–205	185–205	[22]
Ssa197	2	53	131–151	123–179	[23]
Mst85	2	52	149	147–193	[19]
