# Supplementary material for: Genetic Diversity and Conservation of the Prespa Trout in the Balkans
Source: Int J Mol Sci. 2013 Nov 28;14(12):23454–70. doi: 10.3390/ijms141223454 (PMC3876056; doi:10.3390/ijms141223454)
Supplement: Supplementary file 1 [file ijms-14-23454-s001.pdf]

Supplementary Information

Figure S1. *q*-values distribution of the whole sampling genotypes provided by a STRUCTURE run assignment with *k* = 5. G (Golema river system), K (Kranska), BU (Brajcinska upstream), BR (Brajcinska Rzanska) and BD (Brajcinska downstream) samples constitute a subgroup each (in bolt). A (Agios-Germanos river system) and L (lake trout) are composed of various proportions of the 5 former subgroups.

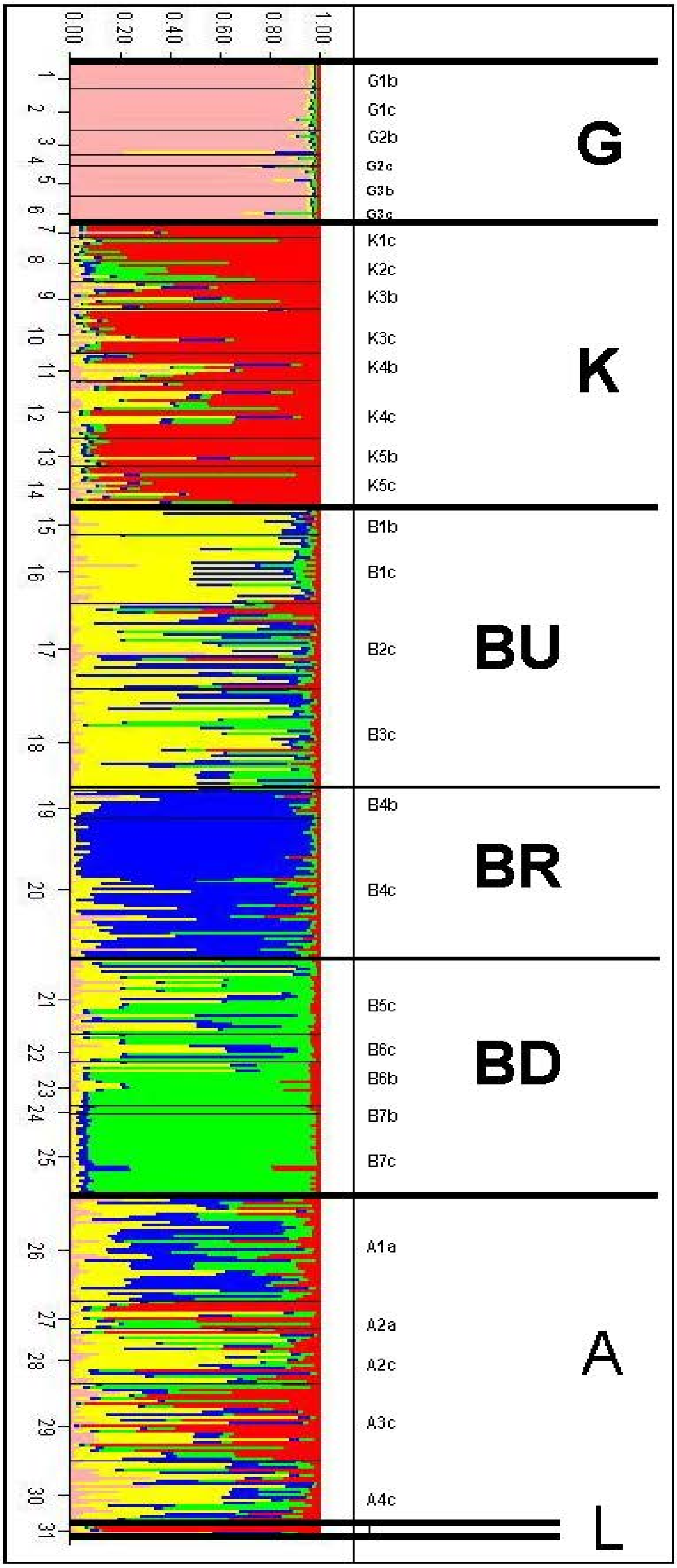

Table S1. List of comparative reference populations used to test the specific nature of the Lake Prespa trout used in Figure 2.

River/Lake	Bassin	Country	Taxon	Haplotype	Date	Sample size
Zi R.	Drini	Albania	*S. trutta*	AD	August 2005	18
Shkumbini R.	Shkumbini	Albania	*S. trutta*	AD	August 2005	20
fish farm (Lake Ohrid)	Drini	Albania	*S. letnica*	AD	August 2005	20
Lake Ohrid	Drini	Albania	*S. ohridanus*	AD	August 2005	20
Bistrica R.	Danube	Slovenia	*S. trutta*	DA	June 1994	16
Trebuscica R.	Soca	Slovenia	*S. trutta*	MA	March 2001	19
Ouveze R.	Rhone	France	*S. trutta*	ME	April 1997	20
Atlantic fish farm	-	France	*S. trutta*	AT	September 2000	20

© 2013 by the authors; licensee MDPI, Basel, Switzerland. This article is an open access article distributed under the terms and conditions of the Creative Commons Attribution license (http://creativecommons.org/licenses/by/3.0/).
